# On Hydrophobia and Tetanus

**Published:** 1813-04

**Authors:** 


*To the Editors of the Medical and Physical Journal.*

GENTLEMEN,

SOME authentic cases of hydrophobia rabiosa,* and tetanus traumaticus,† which have been published lately, make it reasonable to hope, that these two diseases may henceforth be numbered with those which yield to medicine.

Dr. Mackie, of Southampton, in some remarks with which he has introduced his case of tetanus,‡ observes, "that we may trust as fully to opium and the warm bath in the cure of tetanus from wounds, as to mercury in lues, or the Peruvian bark in intermittent fevers." It is surprising that a mode of treatment so perspicuously detailed, and of which the successful issue appears so unequivocally a consequence, should not have become the established practice.

It is now equally certain, that the cure of hydrophobia has been effected chiefly § by excessive bleedings.

But assertions have been added to the detail of the cases of hydrophobia, that an analogy exists between that disease and tetanus; and that the remedies which have been found successful in the latter, may be attended with equal benefit in the former.

I deny the existence of any analogy between these diseases. The classification, by Cullen, of both these disorders, under the order Spasmi, has tended to establish a belief of their analogy in the minds of physicians; and, the opinion of analogy once entertained, an analogous mode of treatment suggested itself of course. It is unfortunate, and not disgraceful, to have gone wrong with Cullen. Wherever he is not a justificator, he is an accuser. Great as my veneration is for that author, I cannot persuade myself to admit, on his arbitrary classification, an analogy between diseases that have no resemblance.

If an analogy really existed between these disorders, and

* Cases by Mr. Timon and Dr. Shoolbred, Edin. Med. and Surg. Journal, No. 33.

† Case of Mr. Scratchley, in Dr. Christie's observations, Edin. Med. and Surg. Journal, No. 32; and Mr. Leath's case, London Med. and Phys. Journal, No. 168.

‡ Duncan's Commentaries, vol. 20.

§ Mercury was also used, and, most likely, with benefit. Its service in destroying the effect of certain kinds of morbid animal poisons, and in many inflammatory disorders, is well known. It certainly is not to be inferred that it was unnecessary, still less injurious; and, till it shall be shewn to be so, it should be continued with the bleedings.

the the practice found so successful in hydrophobia, might, even with a fair probability of success, be applied to tetanus; yet it is so important, that no remedy of *untried effect* should, from the weight of the authority by which it is recommended, supersede a mode of treatment, the efficacy of which has been *fully proved*, that I should still have felt myself urged to hint the want of wisdom, in changing certainty for problem. But, when it is my opinion that the belief in the analogy, which alone can give speciousness to an analogous mode of treatment, is totally unfounded, and would draw fatal consequences if it were acted upon; I should compromise a strong sense of duty, if I did not publish my dissent from the position, and the reasons of it.

It will be enough for my purpose to place descriptions of these diseases by the side of each other.

The cause of one is the introduction of a morbid animal poison into the system; the other arises from a lesion of the nerves in any part of the body.

It is hard for me to believe that any person who has witnessed hydrophobia and tetanus, should pronounce them to resemble each other. On comparing the histories of both diseases in authors, no descriptions can be found that are more strongly distinguished. In tetanus a permanent rigidity of the muscles extends from one set to another, till the whole body becomes as inflexible as a statue; and, at last, seizes the heart* itself, when death ensues. When was there ever a case of hydrophobia in which this affection, even in a degree, formed part of the disease?

In hydrophobia rabiosa, the convulsive horror of liquids always perhaps attending the inflammation of the stomach and fauces, is the most prominent symptom of the disease; when was this ever indubitably a symptom in tetanus? †

The

* Dissections of cases of tetanus, Med. and Phys. Journal, No. 127, by Mr. Howship.

† I am aware that Dr. Darwin, in his Zoonomia, speaks of a case of tetanus which he witnessed, that was preceded by hydrophobia; and I have myself seen in tetanus a dislike to liquids so strong, that a word expressive of a hatred of water is not an unappropriate term for it. Difficulty and pain in deglutition, arising from the rigidity of the muscles about the jaw and neck, often accompany tetanus; and the patient has therefore an aversion to liquids. From the same cause he has an equal aversion to solids, and even to speaking. But this aversion is a reasonable conclusion, derived from the experience of pain in previous attempts to swallow. While, in hydrophobia rabiosa, the dread anticipates the pain of any attempt. The name alone of water excites a horror which is intuitive; the sight

The different methods of cure now recommended in these diseases, (the only methods that have been completely successful,) one depletory, the other stimulative, point out an equal difference in the diseases* themselves; and are such as we may suppose would have been employed long ago, if the appearance of each, after death, had been duly studied.

On morbid dissections of the bodies of those who have died of hydrophobia, marks of inflammation, often approaching to gangrene, have appeared in the stomach and other parts; and almost all writers on this disease represent it to be of an inflammatory kind; they often compare it to fevers, in which it is said all the symptoms of hydrophobia have sometimes appeared. Under this opinion of tendency to inflammation, bleeding, even to excess, has been recommended, but not till lately practised.

In tetanus no tendency to inflammation whatever discovers itself, but the disease appears to be wholly spasmodic; the small quantity of blood, advised to be taken away, is to remove the vascular tension, preparatory to the exhibition of powerful stimuli; more probably would be unnecessary, if not injurious.

If my descriptions of these two diseases are correct, and, if the senses may be trusted, I will vouch their correctness, there can be no analogy between hydrophobia and tetanus, if analogy (as I collect from the use of it by the most classical authors) means resemblance.

---

sight of it is intolerable, and the attempt itself followed by furious convulsions that exhaust the patient. The word *hydrophobia* may have produced the confusion that has taken place. It is not too strong to express the aversion to liquids which sometimes attends tetanus, because it is not strong and copious enough to describe that agony of impatience at the mere thought of them, which marks the disease produced by the bite of a rabid animal. A case of tetanus has fallen under my observation, in which the muscles about the neck and jaw were in a rigid state, and the patient used to wave his hand as a sign to request time for preparation to answer or swallow, on being asked a question, or told to take his medicine. A few moments enabled him to make the adjustment necessary to proportion his efforts to the narrowed state of the organs, and then he both spoke and swallowed with little pain, and without any expression of horror. But, if he forgot the altered state of the parts to be employed, a harsh pain attended the attempt; he started, and left the word half pronounced, or threw down the cup with great emotion in the middle of his draught. The pain excited by these sudden attempts, left an impression of terror, which some time was required to wear away, and during which he could not be persuaded to repeat the attempt.

* Tetanus is purely asthenic; while hydrophobia rabiosa appears to be sthenic.

There is then no reason to apply the same remedy indiscriminately to hydrophobia and tetanus; and happily there is as little necessity. We have the corroboration of repeated instances in which the former has yielded to excessive bleedings, in which the use of mercury was joined; the latter to the almost unlimited use of laudanum, together with the warm bath; and it is a duty to continue the use of these distinct remedies in each of those diseases, till the instances of their failure shall exceed those of their success. This failure, I feel confident, will never happen. It is not often, in the whole practice of medicine, that the event can with more decision be pronounced a consequence of the remedy, than in the cases to which I have alluded. With this degree of certainty, all that can be attained in the art, before us, it would be wantonness to risk experiment. M. N.

*London, Feb.* 13, 1813.

